# Pharmacists detecting atrial fibrillation in general practice: a qualitative focus group study

**DOI:** 10.3399/bjgpopen20X101042

**Published:** 2020-06-24

**Authors:** Vilius Savickas, Emma L Veale, Sukvinder K Bhamra, Adrian J Stewart, Alistair Mathie, Sarah Corlett

**Affiliations:** 1 Medway School of Pharmacy, University of Kent, Kent, UK; 2 Cardiology, Medway NHS Foundation Trust, Kent, UK

**Keywords:** atrial fibrillation, mass screening, general practice, primary health care, pharmacists, qualitative research

## Abstract

**Background:**

Atrial fibrillation (AF) affects up to 10% of people aged ≥65 years, yet a third of all cases remain undetected. Practice-based pharmacists are in an ideal position to facilitate opportunistic AF screening, while increasing general practice capacity at a time of workforce crisis.

**Aim:**

To explore the perspectives of three stakeholder groups involved in the ‘Pharmacists Detecting Atrial Fibrillation’ (PDAF) study to elucidate the facilitators and barriers to pharmacist-led AF screening in general practice.

**Design & setting:**

A qualitative study took place, comprising homogeneous focus groups with stakeholders in Kent, UK.

**Method:**

The stakeholder groups — patients, general practice staff (GPS), and clinical pharmacists (CPs) — were recruited using convenience sampling. Audio-recordings were transcribed verbatim and analysed using a deductive Theoretical Domains Framework (TDF) approach.

**Results:**

Twenty-five patients, four pharmacists, and nine practice staff participated in six focus groups. Three main themes were identified: knowledge and awareness; prioritisation of resources; and environmental considerations. The public’s lack of awareness of AF-related risks and pharmacist-led screening services was highlighted. Practice-based pharmacists were perceived as an underutilised educational resource which, together with novel electrocardiogram devices, enabled convenient access to screening while reducing GPs’ workload. Participants agreed that AF screening should be incorporated into personalised health checks and at-risk groups should be prioritised, such as care home residents. Patients favoured the general practice environment over the community pharmacy where concerns of privacy, staffing, and commercialisation were raised.

**Conclusion:**

The findings of this study support the introduction of pharmacist-led AF screening programmes in general practice surgeries. Commissioners should consider the added value of utilising CPs and focus on the delivery of AF screening within an integrated service.

## How this fits in

AF is a major cause of preventable stroke, which, despite international recommendations for opportunistic screening, remains widely undiagnosed. Primary care network integration of practice-based pharmacists provides a multidisciplinary option for the development of the national AF screening programme during the time of increased general practice workload pressures. This qualitative evaluation of the multi-site PDAF study in Kent (UK) offers an insight into key enablers and barriers to service development from the perspectives of patients, pharmacists, and practice staff. In the absence of published literature pertaining to pharmacist-led AF screening in general practice, the findings presented here provide the necessary evidence in support of the service, while discussing its positive impact for patients and clinicians alike.

## Introduction

AF affects up to one in 10 people aged ≥65 years in England; although, 30% of all cases remain undiagnosed.^1^ Individuals with untreated AF display a five-fold greater risk of cardioembolic stroke, resulting in a preventable annual NHS bill of £2.2 billion.^2,3^ Opportunistic AF screening is recommended by both international guidelines and a 2018 white paper,^4,5^ but is not supported by UK national guidance.^6,7^ Despite this discordance, the government has set a target to detect 85% of AF cases by 2029.^8^

To facilitate AF detection, >6000 mobile single-lead electrocardiogram (ECG) devices have been distributed to primary care settings.^9^ Such devices offer a rapid, convenient, and highly accurate means of AF detection.^10–14^ However, service pressures within general practice have curtailed the implementation of screening services^15,16^ and encouraged commissioners to search for alternative multidisciplinary models of care.^17,18^ The feasibility of pharmacist-led AF screening in community pharmacies has been investigated.^19,20^ Despite promising findings, real-life implementation of the AF screening service in this setting is limited by multiple barriers, including inadequate follow-up.^21,22^

Integration of pharmacists within surgeries may overcome these hurdles and simultaneously increase general practice capacity.^23^ Launched in 2015, the ‘Clinical Pharmacists in General Practice’ pilot created >1500 pharmacist vacancies in general practice surgeries to alleviate GP workload by reviewing patients with long-term illnesses or managing common ailments.^24^ Primary care networks plan to build on the success of this pilot by employing at least one clinical pharmacist (CP) per practice^25^ by 2024 and their roles could include routine screening services.

PDAF was a multi-site study in UK general practice surgeries, which determined the impact of CP-led AF screening using either conventional pulse palpation or novel single-lead ECG devices during the influenza vaccination season (reported elsewhere).^26,27^ This study constitutes a qualitative stakeholder evaluation of the PDAF intervention,^28^ and identifies facilitators and barriers to its implementation focusing on the novel role of pharmacists in general practice.

## Method

Focus groups were conducted to ascertain the perspectives of three stakeholder groups: patients, GPS, and CPs.^26^ This method is commonly used in qualitative research and generates rich data to shape complex healthcare interventions.^29,30^ Stakeholders were recruited using convenience sampling, and all interested individuals were invited to participate.

CPs provided all PDAF participants with an invitation and information leaflet for the focus group regardless of their AF screening result or demographic characteristics. All CPs involved in the PDAF initiative were emailed an invitation to participate at the end of the study by the research team. The gatekeeper at each participating surgery distributed internal email invitations to all GPS. Written informed consent was obtained.

Semi-structured topic guides for each participant group contained open-ended questions and were developed from the literature.^13,21^ All interviews were audio-recorded, transcribed verbatim, coded, and analysed by one research team member. Another researcher independently verified the accuracy of transcription and rigour of data analysis. Both researchers were registered pharmacists and maintained a reflexive account to acknowledge the possible influence of their professional background.^31^

**Figure 1.**
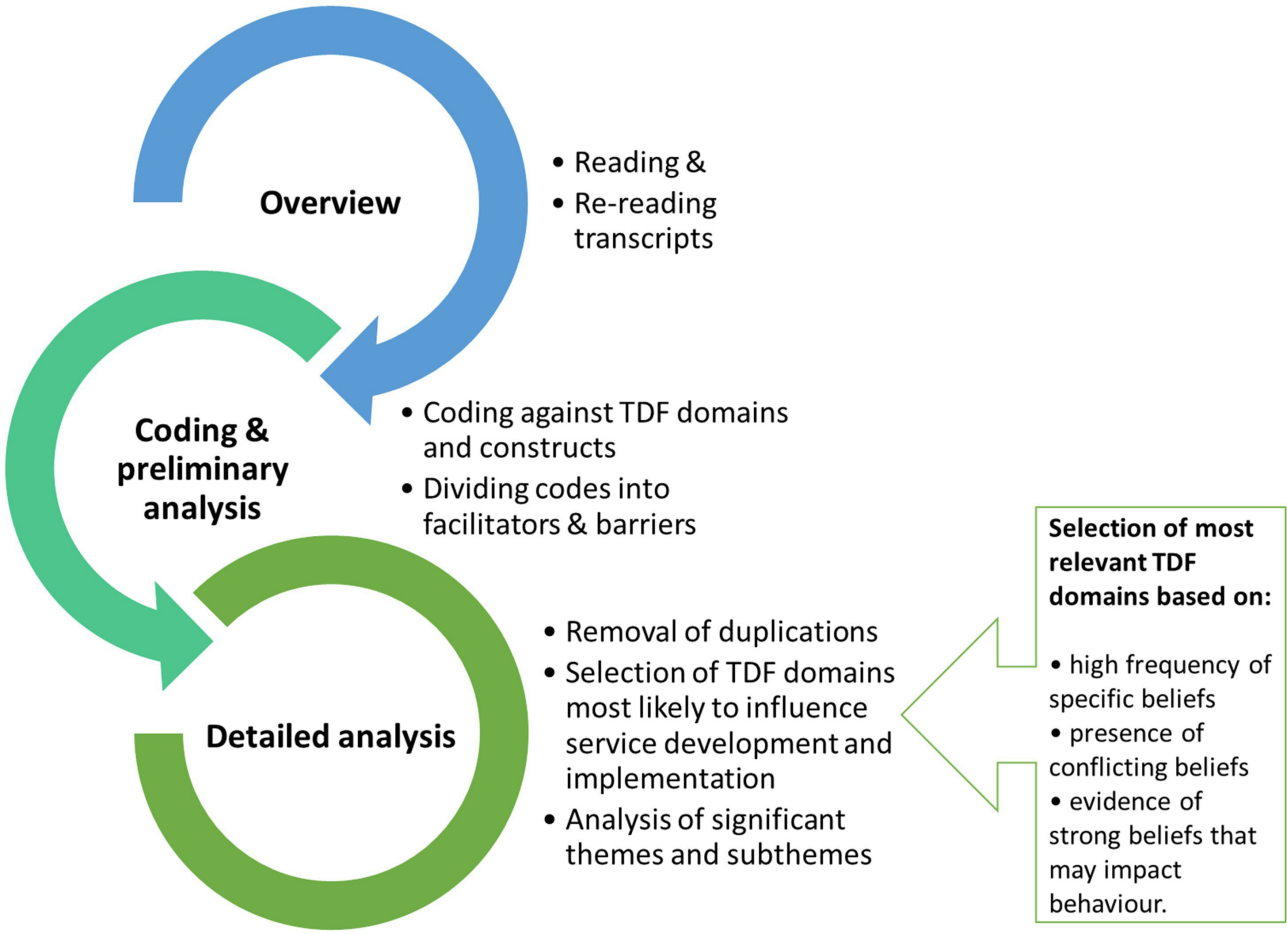
Three-step approach to data analysis based on the Theoretical Domains Framework (TDF) and analytical strategy adapted from Atkins *et al* ^32^ and Islam *et a* *l* ^34^

Patient demographics were analysed using SPSS (version 25). Qualitative data were analysed in NVivo (version 12) using the deductive TDF approach, as detailed in Figure 1.^32,33^ TDF domains most likely to influence the service proposed were selected using the criteria adapted from Islam *et al*.^34^ The major themes and sub-themes within these domains were selected for final analysis of key facilitators and barriers. Deviant case analysis was performed to ensure that perspectives that diverged from dominant trends were not overlooked.^35^

## Results

Twenty-five patients attended four 80–90-minute focus group discussions in January 2018 and February 2019 (5–7 patients per group). Participants from all four practices involved in the PDAF study were represented and were slightly younger than the main cohort (Table 1). Most patients were aware of AF screening taking place and pre-booked their appointments (68%); others were screened before or after their influenza vaccination, or at another appointment (32%).

**Table 1.** Comparison of focus group and PDAF study participants’ demographic characteristics (*n* = 25 and *n* = 604, respectively). Continuous variables are expressed as a median (interquartile range). Categorical variables are expressed as a number (percentage).

	Focus groupparticipants	All PDAF participants
Age, years	71 (68 to 73)^a^	73 (69 to 78)^a^
Male	13 (52.0)	258 (42.7)
**Ethnic** **group**		
White British	23 (92.0)	585 (96.9)
Other	2 (8.0)	19 (3.1)

^a^ *P* = 0.023 as determined by Wilcoxon’s signed rank test.

PDAF Pharmacists Detecting Atrial Fibrillation

Four CPs and nine GPS participated in two separate 40-minute focus groups. CPs had 6–15 years of professional experience, and two of them were male. All GPS were female and worked at one of the four surgeries: three were office support staff, two receptionists, and the remainder a research administrator, a prescribing technician, a student nurse, and a healthcare assistant.

**Figure 2.**
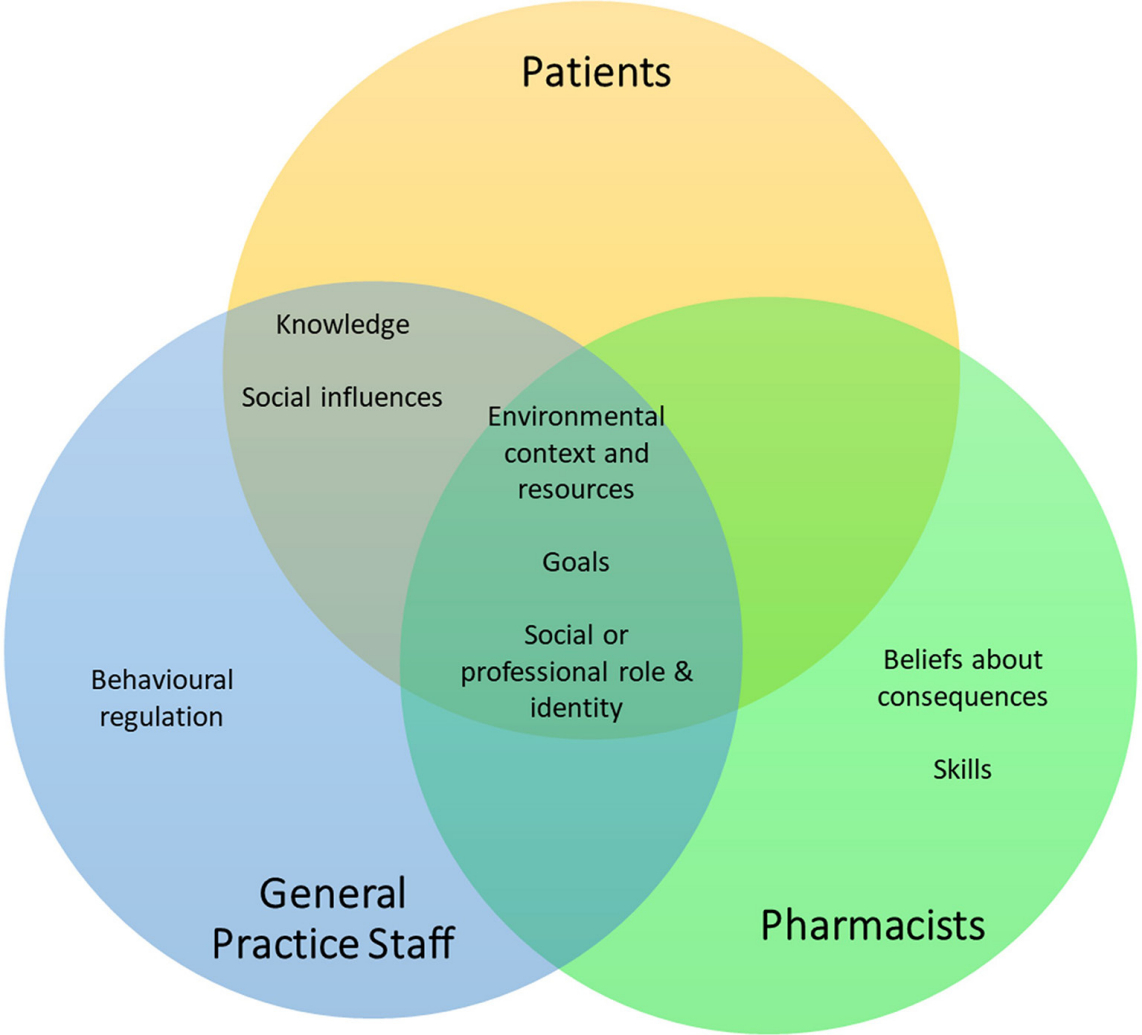
A Venn diagram depicting the TDF domains most likely to influence the facilitators and barriers to service development and implementation identified during the coding and preliminary analysis. The most relevant domains for each stakeholder group were selected using the criteria by Islam *et al* ^34^ (*n* = 25 for patients, *n* = 9 for general practice staff, and *n* = 4 for pharmacists).

Themes from coding and preliminary analysis are summarised in Figure 2. Subsequent analysis identified three overarching themes: knowledge and awareness; prioritisation of resources; and environmental considerations. These themes were mapped onto five most relevant TDF domains (Table 2).

**Table 2.** Key facilitators and barriers to atrial fibrillation screening service proposed mapped against the most relevant TDF domains (*n* = 25 for patients, *n* = 9 for GPS and *n* = 4 for pharmacists).

**TDF domain(s)**	Facilitators	**Stakeholder group(s)**	**Barriers**	**Stakeholder group(s)**
Environmental context and resources	Space and established general practice infrastructure	All	Busy clinic environment	All
	Advantages of single-lead ECG	Patients and pharmacists	Accessibility of community pharmacy	Pharmacists and patients
	Presence of HCP	Patients	Service costs and resources	Patients and GPS
			Variation in practice culture and poor service integration	Pharmacists
			Variable access to care	Patients
			Logistics of same-day screening	
Goals	Prioritisation of at-risk groups	All	Screening led by other HCPs	Pharmacists and patients
	Flexible choice of appointment	Patients and GPS	Self-testing technology	Patients
	Engagement of stakeholders	Pharmacists		
Social or professional role and identity	Utilisation of pharmacists’ skills	All	Misconceptions about pharmacists	Patients and GPS
	Development of pharmacists’ roles	Pharmacists	Unconventional role of pharmacists	Pharmacists
Knowledge and social influences*	Knowledge and awareness	Patients and GPS	Getting used to novel screening	Patients
	Staff inclusion in service provision	GPS	Lack of communication with staff	GPS

*Knowledge and social influences are two separate domains but are combined in this table because some facilitators/barriers mapped onto both domains.

ECG electrocardiogram GPS general practice staff HCP healthcare professional

### Knowledge and awareness

#### Awareness of AF and screening

Patients and GPS admitted that they did not know much about AF-related risks prior to the screening initiative, despite some suffering from other cardiovascular conditions:

‘*Although I’ve had hypertension for 25 years … I wasn’t particularly aware of this other than from our friend who had irregular heart beat, well I thought it’s just an irregular heart beat, similar things.’* (PT9)

Two patients with a pre-study diagnosis of AF emphasised the need to educate the public about the condition and related risks:

*‘Do people know? I didn’t know anything about atrial fibrillation until the age of 63. We all know about breast cancer and colon cancer, AIDS and all kinds of other things where there’s been promotion for people that need testing.’* (PT7)

Patients were motivated to attend owing to personal risk factors, such as older age, family history of heart disease, or social responsibility:

*‘We need to take pressure away from hospitals and, as I said earlier, prevention is better than cure. If you know you’ve got a problem, you can have it treated at point A. You’re not going to end up in point E where you’re gonna spend three or four weeks in hospital.’* (PT19)

All stakeholders agreed that the PDAF study was biased towards proactive, lower-risk patients and that the asymptomatic nature of AF was a particular challenge in engaging less motivated individuals.

*‘People who don’t attend the flu vaccine are probably ones who are more at risk because they’re not looking after their health.’* (CP4)

Patients and staff proposed ways to raise public awareness of AF and improve uptake of screening, including patient-friendly posters, websites, text messages, emails, mobile-phone applications, AF awareness campaigns, and TV and/or radio programmes:

*'Website, leaflets, through to the maybe at-risk patients or things like that.’* (GPS5)

*‘Could we put up posters of Age Concern? In our surgery could we send texts?* *’* (GPS9)

#### Role of pharmacists

Both patients and GPS felt that the public often perceived pharmacists as 'shop assistants' rather than healthcare professionals (HCPs) who can play a role in public health services:

*‘Then people need to be made aware of what the pharmacists can do. Because as far as I’m concerned, the pharmacist is just a guy in a local shop and I go seem him if I’ve got a headache or a cold or something like that.’* (PT3)

A few patients and staff expressed doubts regarding pharmacists’ clinical abilities. Engagement in AF screening appeared to modify patients’ views about CPs, and several patients pointed out that public awareness of pharmacist-led services could be raised by carrying out similar initiatives:

*'And it’s only recently that it’s been done with you, that you actually recognise that a pharmacist is a very, very skilled and trained person and has got an immense knowledge of a wide range of problems …’* (PT14)

### Prioritisation of resources

#### Effective use of novel technology

Patients were fascinated by the mobile technology, which made the AF screening process quick, non-invasive, and painless. They were intrigued by the live recording of their ECG and appreciated the presence of the pharmacist, who provided them with immediate reassurance:

*‘The guy that was talking to us said, “do you realise that your heart works in more than just one way?” and when our recording came out with all these various times, and saying, “this is this part of it working, this is this…” and that fascinated me the fact you go along usually and someone says you know, “right, here’s your heart and it goes bleep, yeah, it’s all fine.”’* (PT14)

In turn, CPs reported patients’ interest in the technology and emphasised the convenience of having a 'pocket' device with them at all times. They expressed a strong 'faith' in the device, which in addition to AF, helped identify other suspected hearth rhythm disorders and was more reliable than conventional pulse palpation:

*‘I just found there was so much variability that actually that’s why I did like the device was because having taken a lot of pulses now, you can see how things could get missed if you just rely on pulses.’* (CP4)

Considering the device’s simplicity, several patients proposed self-testing for AF, questioning whether or not a HCP was required. However, most remained cautious towards the use of technology owing to fear of misdiagnosis:

*‘The danger of doing things in that mind is that you might think you’ve got something very wrong and panic and so on because of what you consider your findings on your computer.’* (PT15)

#### Service costs and resources

Patients generally considered opportunistic AF screening to be worthwhile as part of the broader preventive healthcare agenda. Some were more sceptical, wondering if the screening programme would result in substantial savings when compared with usual care:

*‘If you’re notifying people, radio, TV, whatever, and you’re hoping to identify lots of people who are potentially gonna get or have got AF, and then you can start giving them pills from a certain point, how does that stack up against the cost if you do nothing and then they go into AF and need to be hospitalised?’* (PT6)

Some of these concerns were mentioned by GPS who identified equipment, staff, and follow-up costs as barriers:

*'If it’s funded, then probably* [laughs]*. But I don’t know if it wasn’t because like they were saying all the equipment is going to cost money …’* (GPS1)

*'As well if the pharmacist were provided …’* (GPS3)

‘*And the time because you would have to follow them up so it would be a lot more for you, wouldn’t it?’* (GPS6)

Patients and surgery staff also reflected on the extra resources associated with same-day pre- or post-influenza vaccination screening, such as the waiting time or unplanned parking costs. For others, the efficiency and convenience of same-day screening seemed to counteract the poor use of resources. Three interviewees proposed a flexible system, giving individuals the option to wait or return for an appointment:

*‘Some patients are not gonna come back, you gonna need to grab them when they’re here. Unfortunately, that’s the way they are. But other people will be prepared to come back to a clinic.’* (GPS3)

Aside from time and monetary considerations, CPs and GPS touched on the benefits of stakeholder engagement in AF screening, focusing on GPs, clinical specialists, commissioners, allied HCPs, and administrative staff:

*‘But it works better when the GPs in the vaccination clinic said this would be a good thing done, get it done […] If the GPs didn’t back it up then there was less up take.’* (CP4)

#### Targeting high-risk groups

Numerous patients and pharmacists suggested targeting individuals for screening in public locations, such as supermarkets, gyms or the high street:

‘… *we used to stand there and drag the people off the street to have their blood pressure checked and some of them were an immediate, “Tom, we have to send you to hospital.”’* (PT11)

Pharmacists and GPS spoke about patients who were housebound or care home residents who had limited access to health care, despite being at-risk of cardiovascular disease:

*‘Obviously, you’re missing all of the housebound patients as well because we don’t go to search in care homes, there’s gonna be actually quite a few in care homes.’* (GPS6)

*‘They’re not going anywhere so you’ve got a captive audience.’* (CP3)

Multiple patients thought that the eligibility criteria for AF screening should be broadened to include other at-risk groups, for instance, overweight individuals. Pharmacists and GPS suggested that AF screening could be extended to patients with long-term illnesses such as diabetes or hypertension. The outcome of such discussions was the concept of a personalised health screening plan repeatedly referred to by patients as the ‘MOT’ (a reference to the annual UK Ministry of Transport check for motor vehicles):

*‘If your car is over a certain age and every year you go and have an MOT, then possibly we ought to be doing, thinking the same way …’* (PT14)

#### Pharmacists as underutilised resource

GPS and patients viewed pharmacists as highly qualified practitioners whose expertise was underutilised at the time of increased service pressures, highlighting the juxtaposition of this viewpoint with the widely held perception of pharmacists as 'shopkeepers'. Patients believed that additional pharmacist-led services would reduce GP workload and may improve their access to health care regardless of whether these were delivered in community pharmacies or GP surgeries:

*‘The clinicians say they are terribly overstretched and anything that you can do … And you are not stupid, you are well-qualified people who have a good understanding certainly of pharmacology and medicines. Very well-qualified to do such things.’* (PT1)

As members of the multidisciplinary team, pharmacists were identified as the HCPs to bridge the knowledge gap between patients and doctors:

*‘I think, they kind of act as the middle ground between the GPs and the patients.’* (GPS6)

Reflecting on trust placed in them by patients and staff, pharmacists displayed optimism about the AF screening role, particularly their ability to communicate test results and educate patients. These skills seemed to be the distinguishing point between the pharmacists and technical personnel:

*‘The feedback I got instantly from people was like, “oh wow that’s brilliant you know. That’s really given me some extra information about my, a potential condition that I don’t have or I do have.”’* (CP1)

#### Other HCPs

Patients spoke about utilising other HCPs, for instance, nurses or opticians who may be more accessible than GPs and less costly, yet perceived as more trustworthy than pharmacists. Nurse practitioners with a clinical interpretive ability were identified as being equally as expensive as CPs:

*‘The reason I mentioned* [Opticians X] *is that they’ve extended what they do into hearing, and they do employ professionally qualified people obviously, in both of those areas. I think I trust what they are doing more than the retailer.’* (PT21)

*‘We are more expensive than some nurses, but I think the nurses who feel competent to interpret a 12-lead ECG are probably going to be comparable in cost to some of the pharmacists.’* (CP4)

### Environmental considerations

#### General practice

The majority of patients spoke favourably about the relaxed environment within the consultation. Participants felt free from anxiety and 'mental block', which could occur with traditional health check-ups:

*‘… went into the hospital having my pre-tests and they took my blood pressure, and said, “go on”, it was rocketingly sky high. And then referred back to my GP, GP said, “I suspect this was the scenario”, took my blood pressure and said, “it’s perfectly ok.” And, you know, went in for this … and there’s no anxiety, the difference that you get in the recording is huge. So, we walked away actually feeling quite satisfied.*' (PT14)

However, a few patients, GPS, and one pharmacist complained about the co-running of screening with busy influenza vaccination clinics, which prevented CPs from providing comprehensive pre-appointment information:

‘*Flu clinics are just busy and people are in a big queue … They are coming in and out quickly so to give them a bit of advanced warning or literature might be good.*’ (GPS5)

Pharmacists identified that practice culture and infrastructure influenced how effectively the service was integrated:

*‘One of the health centres was less welcoming and less set up for us to be there, um, the other one was much more accommodating and although you were made to feel quite welcome when we were there outside of the vaccination clinics I felt a little bit more like I was, just kind of visiting* [laughs] *rather than part of the scheme … And the* [Town A] *one was again a little bit ad hoc and the room we had wasn’t ideal.’* (CP4)

Patients debated the ongoing staff shortages, convoluted referral system, and excessively long appointment waiting times, which made general practice widely inaccessible:

‘*This was early December, I’m still waiting for it. Every time I phone up, well the first time I phoned up, they said, “we are full for the next 3 weeks,” which took me to when I was going on holiday. And I phoned them when I came back, and oh yes, and they couldn’t, their diary wouldn’t run that far ahead.’* (PT8)

#### Community pharmacy

Community pharmacies were considered to be more accessible than general practice surgeries, making AF screening in this setting a viable alternative. One participant argued that a close relationship with a local community pharmacist may be more beneficial to their health than engagement with HCPs at the surgery:

*‘And I think that if you build up a relationship with the local pharmacist, you are going to be in a more stable condition than visiting a panel practice which I think a lot of us are, when you rarely see the same doctor two days running or two visits running I should say. And they spend half of their 10-minute slot reading your notes and then they say, “well time’s up, thank you very much, next patient, please.”’* (PT5)

Despite community pharmacy’s accessibility, participants viewed pharmacies as lacking clinical infrastructure or physical space to conduct AF screening consultations. A few patients were concerned about community pharmacy’s commercial nature and their confidentiality:

‘*The one thing psychologically against going to pharmacist is that basically, my one is in Pharmacy V* [large pharmacy chain] *or there’s one near the surgery, they are like shops. You don’t think, “oh, well if they ask me to do something, you know, is it going to be here in front of people buying their soap?"’* (PT8)

Apart from space considerations, some participants doubted that a typical community pharmacy had sufficient staff to facilitate public health initiatives in addition to their traditional supply function:

*‘It wouldn’t work because of CP2’s point, is that even with accuracy checking technicians, you still need a screening of the prescription.’* (CP4)

The waiting areas of general practice were thought to provide HCPs with enough time to approach eligible patients in contrast to the busy community pharmacy environment:

*‘If you were to do it in a local pharmacy, how would you identify people that you wanted? Because our pharmacy is very busy. People come in there all the time whereas if you were at the surgery, there’s people sitting and waiting and you can sort of observe the type of person you’re looking for perhaps.’* (PT3)

## Discussion

### Summary

Facilitators and barriers to novel AF screening services emerged from interviews with three stakeholder groups, predominantly within five TDF domains. Patients and staff highlighted the necessity to raise public awareness of AF-related risks and the clinical roles of pharmacists. Most interviewees were fascinated by single-lead ECG technology, and identified pharmacists as qualified but underutilised practitioners who could increase GP capacity at the time of a workforce crisis. Pharmacists welcomed the evolution of a new clinical role focusing on their advisory and educational skills. Despite superior accessibility of community pharmacy, patients preferred pharmacist-led AF screening in general practice surgeries, which were viewed as more established and less commercialised. All stakeholder groups agreed on the need to develop AF screening programmes prioritising at-risk individuals, such as those attending diabetes or hypertension clinics, or those residing in care homes.

### Strengths and limitations

The method of focus group discussion provided a key advantage of interaction between the members of each stakeholder cohort. Patients were demographically representative of the PDAF study sample and displayed both concordant and diverging opinions on different aspects of service design. This study also benefitted from the group interview with GPS, the majority of whom were not directly involved in PDAF and were, therefore, able to provide impartial views.

The convenience sampling strategy may have overlooked those with limited interest in or access to healthcare initiatives. Most patients were also registered at a single surgery, and it is possible that their opinions influenced the themes derived, despite the facilitators’ attempts to take the perspectives of all participants into account. Lack of GP or senior manager participation in this study was another limitation, although it may have had a positive impact by minimising the influence of any hierarchical relationships.^36^

### Comparison with existing literature

Research studies in Australia have identified lack of public awareness of AF as the primary barrier to patient engagement in screening initiatives.^13,22^ Stakeholder perspectives presented here conformed with such findings. The combined views of patients and staff suggested that this barrier may only be overcome through the delivery of a structured, multifaceted programme, which resembled the 'layered' approaches by Lowres *et al* ^21^ or Sabater-Hernández *et al*.^22^ The closer engagement of stakeholders could be encouraged through central leadership of GPs, practice managers, and commissioners, perhaps by allocating a responsible person previously referred to as a 'designated champion'.^14^

As expected,^22^ despite their interest in technology, patients were unenthusiastic about self-monitoring, praising the advisory value of pharmacists. For example, CPs may be able to reassure the patients with suspected AF or inconclusive diagnoses who could otherwise experience anxiety after a false positive test result at home.^37^ Practice nurses were considered a possible substitute to pharmacists and had previously demonstrated a high degree of confidence in carrying out AF screening.^13,14^ In a recent survey however, only 25% of nurse practitioners felt qualified to make decisions about AF management post-ECG.^16^ This is an area where CPs may use their medicines expertise, for instance, by developing one-stop AF screening and anticoagulation clinics within an established clinician-referral pathway.^38,39^

Similar to discussion by Orchard *et al*,^14^ the combination of AF screening and influenza vaccinations during the PDAF initiative may have engaged the ‘annual’ surgery visitors. Nevertheless, this group appeared to be the proactive, lower-risk ‘healthy volunteers’^40^ rather than the hard-to-reach, at-risk group such as care home residents.^41,42^ As an alternative, an ‘MOT’ screening package was proposed to target AF and related comorbidities of diabetes and hypertension, focusing on at-risk patients; a variation of the community pharmacist-led programme by Twigg *et al*.^20^

During the present study, interviewees were not eager to pursue AF screening within the 'shop' environment of community pharmacies and preferred the less accessible but more 'trusted' general practice surgeries. Interestingly, public perception was altered on pharmacists' integration within surgery environments, where they were regarded as competent HCPs. This environment–identity interaction is not uncommon, but appears to fade over time as pharmacists transition into the new practice role.^43,44^

### Implications for research and practice

Practice-based CPs provide a wide range of clinical services^45^ and have become an integral part of the NHS Long-term Plan.^18,25^ Data presented here suggests that, as experts of medicines and public health, these professionals are ideally placed to conduct AF screening, to educate the public and to address medicines-related concerns as part of a cardiovascular ‘MOT’ service. It is likely that the specification of this holistic service will evolve from the government’s cardiovascular agenda,^8^ while targeting medicines optimisation among those at risk of AF, such as patients with type 2 diabetes.^46^

Regardless of the approach, it should make use of evidence generated through ongoing national AF screening efforts^9^ and the additional general practice pharmacy workforce, which had been demonstrated to produce cost-savings and to free up GPs to focus on more complex patients.^23,47,48^ Stakeholders interviewed here suggested that pharmacists might help utilise the strengths of existing practice infrastructure and clinical expertise, while addressing the major barrier of inaccessibility. Future AF screening guidelines may also wish to consider the favourable profile of single-lead ECG devices highlighted by both patients and providers.

A separate evaluation of PDAF screening results and cost-effectiveness is expected to provide further evidence to support the qualitative perspectives of a novel pharmacist-led service discussed here. A future research programme will also aim to capture the views of GPs by conducting semi-structured interviews with those who may or may not be involved in the delivery of AF screening services.
